# Focus on the Social Aspect of Autism

**DOI:** 10.1007/s10803-017-3407-7

**Published:** 2017-11-29

**Authors:** Joanna Kałużna-Czaplińska, Ewa Żurawicz, Jagoda Jóźwik-Pruska

**Affiliations:** 1 Department of Chemistry, Institute of General and Ecological Chemistry, Lodz University of Technology, Żeromskiego 116 Street, 90-924 Lodz, Poland; 2 CONEM Poland Chemistry and Nutrition Research Group, Lodz, Poland

**Keywords:** Autism spectrum disorder, Prevalence, Expenditures, Costs

## Abstract

Autism spectrum disorder (ASD) describes a set of neurodevelopmental disorders. Despite extensive ASD research lasting more than 60 years, its causes are still unknown. Without indicating the etiology, its development cannot be stopped. Over the years, both the definition and diagnostic criteria have developed. The number of ASD incidence is rising. The economical aspect should also be highlighted. This disorder presents particular challenges to affected children, their parents and physicians. The research of ASD, physician activities, policy making and raising the level of awareness must be coordinated. Special attention should be paid to the problem among adults with ASD.

## Introduction

The more we understand autism spectrum disorder and how it affects patients and their families, the easier it is to determine the support and resources required by each individual case. To understand what ASD is, in the current approach we should refer to the Diagnostic and Statistical Manual of Mental Disorders, 5th edition (DSM-5). Autism spectrum disorder is a set of neurodevelopmental disorders, which includes autistic disorder, Asperger’s disorder and pervasive developmental disorder. It is characterized by the presence of deficits in social communication and social interaction across multiple contexts, and restricted, repetitive patterns of behavior, interests, or activities (APA 2013). Diagnosis of ASD is based on comprehensive behavioral evaluations. Despite numerous studies, the etiology of ASD is unknown and remains the subject of speculations and misconceptions. Nevertheless, there is evidence of a strong influence of genes on the pathogenesis of ASD. Twin studies show that if one of a pair of identical twins meets the diagnostic criteria for ASD, the probability that the other will have the disorder is as high as 58% for boys and 60% for girls, while in the case of fraternal twins the probability falls to 21% for boys and 27% for girls (Hallmayer et al. 2011). In order to get a thorough understanding of causes of ASD, numerous factors and their interactions have been examined (Szatmari et al. 2012), including a number of genetic disorders (Xu et al. 2012), anatomical brain abnormalities (cerebellum, brain stem, hippocampus, amygdale, and frontal lobes) (Belmonte et al. 2004) and the comorbidities such as psychiatric, neurological, metabolic and gastrological disorders (Żurawicz et al. 2013; Kałużna-Czaplińska et al. 2013, 2014; McElhanon et al. 2014).

Literature reports that parents of children with ASD experience increased psychological distress, including depression, anxiety, obsession-compulsion, interpersonal sensitivity, hostility, schizoid traits, paranoia, and schizophrenia (Karst and Van Hecke 2012). Stressors refer to both family and social life and include emotional aspects of having a child with a disability, understanding a child’s needs, various aspects of primary care, medical and education services as well as financial hardship (Gupta and Singhal 2005; Tehee et al. 2009). Because of the fact that children of different racial, socioeconomic and ethnic groups are affected by ASD and due to the scale of its prevalence, many problems related to ASD pose a challenge to the whole of society.

The aim of this paper was to present that the increasing prevalence of ASD has given this disorder a social dimension. Economic costs and other day-to-day problems of ASD patients and their families were described.

### Prevalence and Social Aspect of ASD

Epidemiological surveys provide the basis for awareness, planning policy and service needs, and offer the information necessary to identify potential risk factors for ASD. They also initiate the investigation of ASD prevalence in countries where such estimations have not yet been conducted. The earliest epidemiologic studies performed in the late 1960s according to Kanner’s criteria indicated the prevalence of autism to be 0.5 per 1000 people among children in England (Lotter 1996). Since then epidemiological studies of autism followed by ASD have been performed in various places worldwide. A summary of these studies is shown in Table 1.

**Table 1:** The prevalence rate of autism and ASD

The study period	Prevalence (per 1000)	Geographical area	Criteria	Male to female ratio	Ref.
1966	0.5	England	Kanner’s criteria	2.6	Lotter (1966)
1966–1998	0.5	12 countries (UK, Germany, USA, Japan, Sweden, Ireland, Canada, France, Indonesia, Norway, Denmark, Iceland)	12 different criteria	3.8	Fombonne (1999)
1987–1994	1.1	California	DSM-III-R, DSM-IV	NR	Croen et al. (2002)
1990–1991	11.6	UK	ICD-10	3.3	Baird et al. (2006)
1994–1996	18.1	Japan	DSM-IV	2.8	Kawamura et al. (2008)
1994–1998	12.0	Iceland	ICD-10	2.8	Saemundsen et al. (2013)
1996	3.4	USA	DSM-IV	4.0	Yeargin-Allsopp et al. (2003)
1996–2002	5.1	Norway	ICD-10	4.3	Isaksen et al. (2012)
1997–2008	4.7	USA	NR		Boyle et al. (2011)
2000	6.7	USA	DSM-IV-TR	2.8–5.5	CDC (2007a)
2002	6.6	USA	DSM-IV-TR	3.4–6.5	CDC (2007b)
2003–2004	15.7	UK	ICD-10	NR	Baron-Cohen et al. (2009)
2005–2009	26.4	South Korea	DSM-IV	2.5	Kim et al. (2011)
2006	0.2	China	ICD-10	2.5	Li et al. (2011)
2006	9.0	USA	DSM-IV-TR	3.2–7.6	CDC (2009)
2007	11.0	USA	DSM-IV	NR	Kogan et al. (2009)
2008	11.3	USA	DSM-IV-TR	2.7–7.2	CDC (2012)
2010	14.7	USA	DSM-IV-TR	4.5	ADDM Network (2014)
2010	6.5	Israel	DSM-IV	5.2	Davidovitch et al. (2013)
2010	8.0	Sweden	ICD-10	4.0	Nygren et al. (2012)
2011–2012	20.0	USA	NR	4.6	Blumberg et al. (2013)
2012	14.6	USA	NR	4.5	CDC (2016a)
2011–2013	12.5	USA	NR	NR	Zablotsky et al. (2015)
2014	22.2	USA	NR	NR	Zablotsky et al. (2015)

Data released in 2016 indicate the prevalence of ASD at 14.6 per 1000 (1 in 68) people among children in the USA. No change was noticed compared to the previous report published in 2014 (CDC 2016b). ASD can be diagnosed by age 2, yet in most children ASD was identified after age 4. Less than half of young patients were diagnosed with ASD by age 3 (ADDM Network 2016).

According to the data, ASD is 1.2 times more likely among white children compared to black children. Additionally, it is more common in non-Hispanic white children (15.5 per 1000) than in non-Hispanic black children (13.2 per 1000) and Hispanic children (10.1 per 1000) (CDC 2012, 2016b; Christensen et al. 2016).

As it is presented in Table 1, the male-to-female prevalence ratio varies widely and ranges from 2.5 (Kim et al. 2011; Li et al. 2011) to 7.6 (CDC 2009). It is not entirely clear how much of the increase in prevalence of ASD can be explained by a true expansion of the incidence or by other factors such as changes in the definition of ASD and diagnostic criteria, variations in research methodology as well as greater awareness and recognition.

A large impact of diagnostic criteria on the increasing rates of ASD is the result of the lack of definitive diagnostic tests for ASD. The diagnostic criteria are changing and developing with the evolution of the definition of ASD (King and Bearman 2009; Fisch 2012). Incompatibilities among diagnostic criteria may affect the interpretation of subgroups of ASD in epidemiological studies. This problem is particularly evident in Asperger’s syndrome, for which the DSM-IV/ICD-10 criteria determine age-appropriate development of language, adaptive skills and curiosity, up to 3 years of age. The application of these criteria will result in significant differences in the diagnosis of Asperger’s syndrome and autistic disorders than in the case of application of Gillberg’s et al. criteria (2001). As it can be seen in Table 1, the application of DSM or ICD diagnostic criteria in studies depends on the geographical region. Therefore, the comparison of the prevalence of ASD in Europe and in the United States can be difficult. There is a potential impact of the new DSM-5 criteria on the ASD prevalence and it is currently under consideration. Significant changes in these criteria include a complete removal of Rett’s disorder and consolidation of autistic disorder, Asperger’s disorder and pervasive developmental disorder into autism spectrum disorder, combination of socialization and communication deficits into one set of symptoms and extending the age criterion (APA 2013; Matson and Kozlowski 2011).

Despite the same diagnostic criteria, time frame and location, the application of a more thorough assessment may double the prevalence estimate within the same population. This may indicate the necessity for a careful selection of the research method (Posserud et al. 2010). The differences in the research procedure may include terms of case-finding, sampling and diagnostic definitions (Baron-Cohen 2009). It has been proved that the level of detail collected about and reviewed on children from multiple sources improves the result of estimates (CDC 2009).

**Fig. 1:**
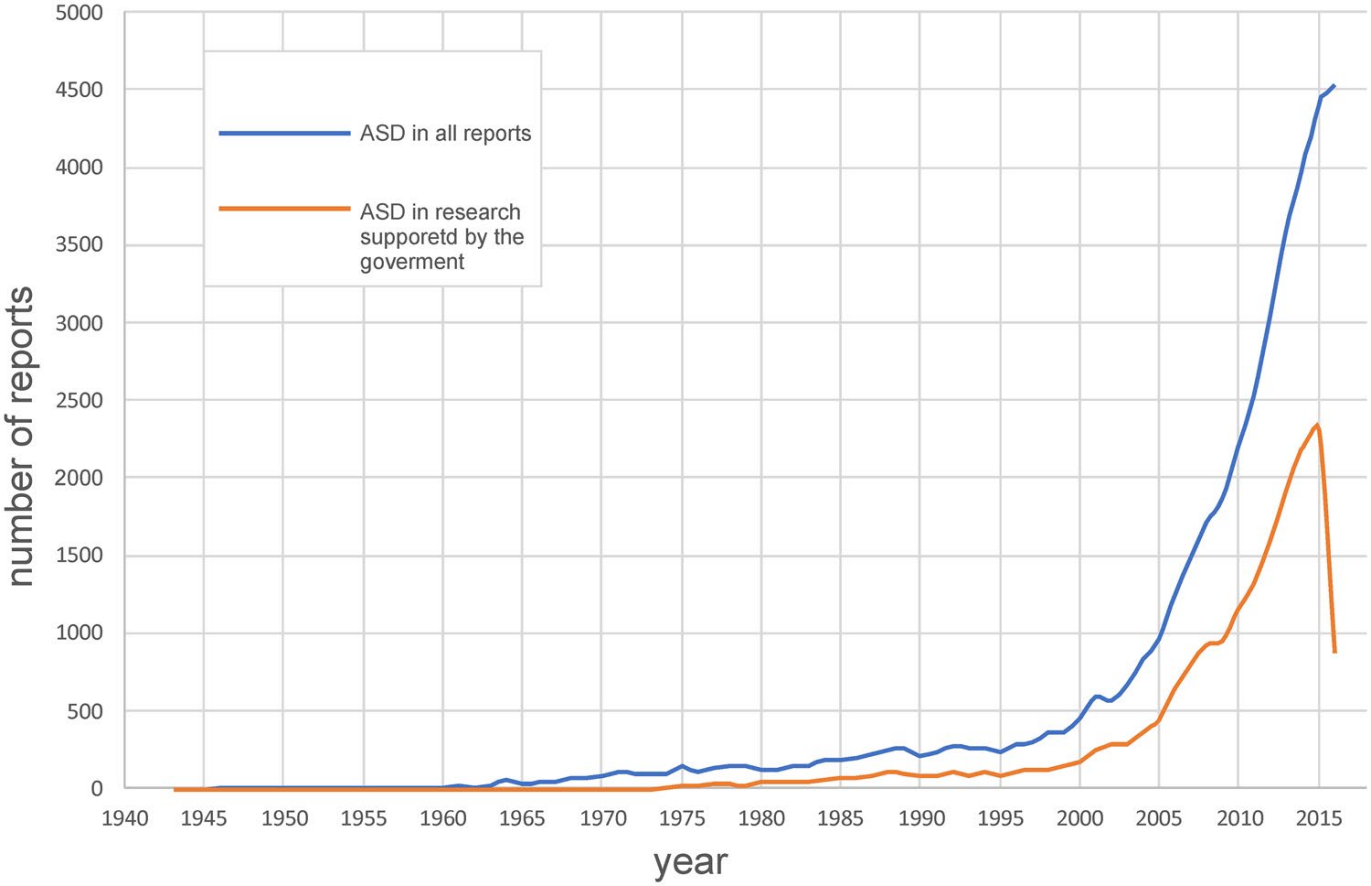
Frequency of reports concerning ASD (1946–2016) Bibliographic research related to the use of term “autism or autistic” in records from the collection of PubMed database. In the search two sets of proceedings: with filters (Article types: Research Support, U.S. Government; Research Support, U.S. Gov’t, P.H.S.; Research Support, U.S. Gov’t, Non-P.H.S.; Research Support, Non-U.S. Gov’t) and without any filter were applied. Results by year (as timeline) were downloaded in the CSV file. Records from 2017 were excluded from the timelines due to the lack of a full annual balance sheet

Considering the changes in the rates of ASD, increased awareness cannot be overlooked. Qualified professionals provide a proper diagnosis in accordance with current criteria. Whereas, increasing knowledge of ASD in a society may lead to a careful observation of disturbed behavior in children by their parents and, consequently, an early search for specialist advice. Their awareness began to rise from the 1960s along with the number of works on the nature of autism (Lotter 1967; Rutter et al. 1970; DeMyer 1975). Nevertheless, until the 1980s autism was too little known to be located in a separate place in any of the ICD or DSM classifications. In1980 the term ‘infantile autism’ was introduced under the scope of a newly generalised category of ‘pervasive developmental disorders’ (PDD) (APA 1980). A significant increase in the knowledge of autism began in the 1990s after the publication of the diagnostic interview schedule Autism Diagnostic Interview-Revised (ADI-R), which was originally used for research purposes (Lord et al. 1994). Higher awareness is reflected in the growing amount of research funding available for ASD and the number of ASD research grants (Singh et al. 2009). Figure 1 shows a rapid increase in both the number of all published reports concerning ASD and research supported by the governments in the last decade.

The role of mass media and all information campaigns conducted by various governmental and non-governmental organizations such as Centers for Disease Control and Prevention (Atlanta, USA), Autism-Europe (Brussels, Belgium) or Action for Autism (New Delhi, India) is very important. Thanks to them, the knowledge about ASD reaches a wide group of people.

### Expenditure on Children with ASD

Due to the high incidence of ASD as well as a wide range of medical services required for affected children, the impact of ASD on the family and public budget is becoming increasingly important. Determination of expenditure related to ASD allows service planning, allocation of resources and appropriate changes in policy (Mandell et al. 2006). Children with ASD have, on average, medical expenditures from 3 (Croen et al. 2006) to even 9 (Mandell et al. 2006; Peng et al. 2009) times higher than other children.

Differences in the expenditure between children with ASD and other children largely come from total outpatient care, physician visits, and medications prescribed (Liptak et al. 2006). The economic aspect can differ even between individuals with ASD and arise from personal needs connected with treatment, care and support. Costs often depend on age of the patients (Buescher et al. 2014). Estimation of costs should also include the ability of parents of ASD children to work. The study has shown that mothers of patients with ASD have lower incomes than mothers of children with no health limitation. They are less likely to be employed and less likely to have full time jobs (Cidav et al. 2012). The reason also lies in the difficulty in finding appropriate and affordable child care (Parish and Cloud 2006).

In estimating the health care costs of children with ASD, the presence of co-occurring conditions should be taken into consideration. Almost half of the children with ASD can have one of the three co-occurring conditions (including attention deficit/hyperactivity disorder (ADHD), intellectual disability (ID) or epilepsy). Therefore, in children with ASD and one or more co-occurring conditions, average expenditures are even twice as high as in those with ASD without a co-occurring condition (Peacock et al. 2012). It was also found that early intensive behavioral intervention for children with ASD affects long-term monetary savings for families and society (Jacobson et al. 1998).

The costs associated with ASD can also directly affect the family of a child with ASD through family out-of-pocket expenses, including home and garden special adaptations, replacement and repair of house and content damages, specialist equipment (e.g. pushchairs and toys), training and seminars courses, travel, diet specialists, education materials. Half of the families of children with ASD report time off work due to their child’s illness over the period of 6 months (Barrett et al. 2012). The average loss of annual income among parents of children with ASD is even 14% of their reported income (Montes and Halterman 2008). A summary of expenditures for children with ASD is presented in Table 2. It should be noted that differences in expenditure among the USA, UK and China may result from differences in practice style and health care financing.

**Table 2:** Expenditure on children with ASD

Country and year for costs	Age	Cost categories taken into account	Study outcome	Ref.
USA (Pennsylvania) (1994–1999)	0–21	Health care costs	$9980 per year	Mandell et al. (2006)
USA (North Dakota) (1998–2004)	0–20	Health care costs	$7894 per year	Peng et al. (2009)
USA (1999–2000)	< 19	Health care costs	$6132 per year	Liptak et al. (2006)
UK (1999–2000)	4–10	Health care costs and non-health care costs	£ 689 per week	Järbrink et al. 2003
USA (2000–2004)	< 18	Health care costs	$5592 per year	Leslie and Martin (2007)
USA (2004)	1–21	Health care costs	$6830 per year	Shimabukuro et al. (2008)
USA (California) (2003–2004)	2–18	Health care costs	$2757 per year	Croen et al. (2006)
USA (2003–2005)	2–17	Health care costs	$10,709 per year	Peacock et al. (2012)
USA (2005)	≤ 15	Loss of income of parents associated with having child with ASD	$6200 per year	Montes and Halterman (2008)
USA (2005)	3–20	Health care costs	$14,957 per year	Cidav et al. (2013)
UK (2006–2007)	2–4	Health care costs and non-health care costs	£ 3083 per 6 month	Barrett et al. (2012)
China (2007–2009)	1–15	Health care costs	RMB 17,293 per year	Wang et al. (2012)
USA (2011)	NR	Health care costs	Higher by $4110–$6200 per year	CDC (2016a)
USA (2011)	NR	Behavioral intervention	$40,000–60,000 per year	CDC (2016a)

While a wide range of papers is focused on the childhood autism, its prevalence, costs and treatment, it is hard to find information about care system of adults with this disorder. The majority of reports refer to patients in the age range 3–17. This tendency clearly indicates how little attention is paid to individuals with ASD.

As ASD is characterized by social, communication and behavioral disabilities, their severity will influence the ability to work and live independently. The growing interest in the use of medical home model for adults with ASD can be seen. Yet, little is known about their availability (Bruder et al. 2012). The health care status of adults with ASD is also neglected and concentrates mainly on the comorbid conditions (Koritsas and Iacono 2009). It is reported that the vast majority of ASD patients is unable to live independently (Bruder et al. 2012).

### Conclusions and Directions for the Future

Autism spectrum disorder has a significant impact on the health care and finance systems and represents a challenge in the research planning. As it is shown in Fig. 1, the number of ASD research grants funded by the USA and non-USA governments in the last decade has significantly increased. In the USA, in the years 1997–2006 the majority of projects concentrated on basic science (65%) compared to clinical (15%) and translational research (20%) (Singh et al. 2009). A very important area of research is an early detection of ASD.

To meet the concerns of adults with ASD, family members, practitioners and researchers, the areas that make a difference to people’s day-to-day lives (e.g. effective public services, evidence-based interventions, programs to enhance individuals’ life skills, understanding of the place of people with ASD in society) should be among the research priorities (Pellicano et al. 2014).

High incidence of ASD increases problems with access to early diagnosis and intervention as well as economic issues associated with ASD, which in turn deepens the family crisis. Major challenges for the future of a child with ASD include education and the possibility of employment in adulthood (Pellicano et al. 2014). All of these problems associated with ASD should find support in the legal system. In order to improve living conditions of children with ASD and their families, there is a need for the development of social policy in four key areas: health insurance, public funding policies which support evidence-based practices for ASD, federal policies and funding which support equal access to services, criteria for evidence-based practices, and research that meets the criteria for evidence-based practices (Howlin and Moss 2012).

The progress observed in the last decade associated with the diagnosis, treatment, awareness, funding, as well as improving the functioning of the family of a child with ASD allows optimism in relation to the future. It should be noted that the research of ASD, physician activities, efforts to develop policies and raising awareness should be coordinated and the ethical issues should be taken into account.

## Abbreviations

ADHD: Attention deficit/hyperactivity disorder
ADI-R: Autism diagnostic interview-revised
ASD: Autism spectrum disorder
DSM: Diagnostic and statistical manual of mental disorders
ICD: International classification of diseases
ID: Intellectual disability
PDD: Pervasive developmental disorders
P.H.S.: Public health service
